# Changes in bispectral index and patient state index during sugammadex reversal of neuromuscular blockade under steady-state sevoflurane anesthesia

**DOI:** 10.1038/s41598-023-31025-9

**Published:** 2023-03-10

**Authors:** Jeayoun Kim, Doyeon Kim, Inho Kim, Ji Seon Jeong

**Affiliations:** 1 Department of Anesthesiology and Pain Medicine, Samsung Medical Center, Sungkyunkwan University School of Medicine, 81 Irwon-Ro, Gangnam-Gu, Seoul, 06351 Korea; 2 Department of Anesthesiology and Pain Medicine, CHA Bundang Medical Center, CHA University School of Medicine, Seongnam, Korea

**Keywords:** Clinical trial design, Clinical trials

## Abstract

Few studies have investigated the changes in patient state index (PSI) and bispectral index (BIS) in response to abrupt increase in electromyographic (EMG) activity. These were performed using intravenous anesthetics or reversal agents for neuromuscular blockade (NMB) other than sugammadex. We compared the changes in BIS and PSI values caused by the sugammadex reversal of NMB during steady-state sevoflurane anesthesia. We enrolled 50 patients with American Society of Anesthesiologists physical status 1 and 2. At the end of the surgery, we administered 2 mg kg^−1^ sugammadex while maintaining sevoflurane for a 10-min study period. The changes in BIS and PSI from baseline (T_0_) to train of four ratio of 90% were not significantly different (median difference 0; 95% CI − 3 to 2; P = 0.83), neither were the changes in BIS and PSI values from T_0_ to their maximum values (median difference 1; 95% CI − 1 to 4; P = 0.53). Maximum BIS and PSI were significantly higher than their baseline values (median difference 6; 95% CI 4–9; P < 0.001 and median difference 5; 95% CI 3–6; P < 0.001, respectively). We found weak positive correlations between BIS and BIS-EMG (r = 0.12, P = 0.01), as well as PSI and PSI-EMG (r = 0.25, P < 0.001). Both PSI and BIS were affected to some extent by EMG artifacts after sugammadex administration.

## Introduction

Several electroencephalogram (EEG)-based parameters were developed to assess anesthetic depth to prevent anesthesia awareness or overdosing of anesthetic agents^1^. The bispectral index (BIS) monitor (BIS, Medtronic, Minneapolis, MN, USA) is a traditional global market leader^2^. BIS monitor analyzes a one or two-channel EEG from a single hemisphere and uses power and phase information between distinct EEG frequency bands, α, β, δ, and θ. The BIS has a range from 0 to 100, with decreasing values indicating increasing levels of hypnosis and the range of 40–60 indicates an optimal hypnotic state for general anesthesia.

The SedLine^®^ monitor (Masimo Corp, Irvine, CA, USA) provides patient state index (PSI) as a relatively newer parameter for anesthetic depth based on different proprietary algorithms. PSI sensor extracts EEG signals through channels separate from those of electromyography (EMG). In addition to quantitative EEG analysis of the power within the α, β, δ, and θ frequency bands, it analyzes the spatial and temporal gradients of EEG frequency bands using four individual EEG channels from both hemispheres, which separates the PSI monitor from BIS monitor^3^. The PSI values range from 0 to 100 and the range of 25–50 indicates an optimal hypnotic state for general anesthesia.

The administration of sugammadex, a highly selective γ-cyclodextrin, that promptly reverses monoquaternary steroidal neuromuscular blocking agents is reported to increase BIS value despite continuous administration of anesthetic drugs^4–7^. The most plausible explanation for increased BIS is a sugammadex-induced activation of the frontalis EMG signal, as suggested in previous studies^4–10^. Alternatively, it could be a sign of actual arousal induced by an abrupt increase in afferent signals from the muscle spindle to the arousal center in the central nervous system^8,11,12^.

Differences in proprietary algorithms and data sources may yield different performances in rejecting interference such as EMG and electrocautery. For example, the PSI monitor was less disturbed by electrocautery during surgery than the BIS monitor^13^. In a previous study, we analyzed the changes in BIS and PSI values and observed similar changes during sugammadex reversal of NMB under propofol-remifentanil intravenous anesthesia^14^. However, the performance of BIS as an indicator of anesthetic depth is better in propofol anesthesia than in sevoflurane anesthesia^15^. In contrast, the performance of PSI was consistent regardless of the type of anesthetic agents used^16^. Based on these findings, PSI may be better than BIS in filtering out EMG artifacts during sevoflurane anesthesia. Consequently, PSI may be less affected by the sugammadex reversal of NMB than BIS during sevoflurane anesthesia. Understanding the effects of sugammadex administration on EEG-based parameters of anesthetic depth is critical for ensuring safe and effective anesthesia. Therefore, we aimed to investigate the changes in PSI and BIS and the effect of EMG after sugammadex administration under steady-state sevoflurane anesthesia.

## Methods

### Ethics

The study was approved by the Samsung Medical Center Institutional Review Board (no. SMC 2020-09-175, approval date: 18/11/2020) and registered with the Clinical Trial Registry of Korea (https://cris.nih.go.kr; Date of first registration: 30/12/2020; Principal investigator: Ji Seon Jeong; Registration number: KCT0005722) before recruitment of the first participant. Written informed consent was obtained from all participants before enrollment in this study. All methods were performed in accordance with the Declaration of Helsinki and its revisions.

### Patients and anesthesia

We screened patients scheduled for elective orthopedic surgery under sevoflurane anesthesia to participate in this study. After obtaining written informed consent, we assessed the patients for eligibility. Adult patients, aged 19–70 with American Society of Anesthesiologists physical status I or II, were enrolled between January 2021 and April 2021. Exclusion criteria were as follows: body mass index > 30 or < 18.5 kg m^−2^, emergency surgery, pregnancy or nursing, alcohol or drug abuse, a disease affecting the central nervous system, psychiatric disorder, neuromuscular disease, severe liver disease or kidney disease, significant arrhythmia or cardiovascular disease, and disease for which the use of the study drug was contraindicated because of a history of allergies or hypersensitivity.

We provided no premedication. Standard monitoring was applied including intermittent noninvasive blood pressure, a three-lead electrocardiogram, and peripheral pulse oximetry. After wiping the forehead of the patient with 70% alcohol and allowing it to dry, a PSI sensor (RD SedLine EEG Sensor; Masimo Corp., Irvine, CA, USA) and a BIS Quatro electrode (BIS Quatro Sensors XP; Medtronic, Minneapolis, MN, USA) were attached to the forehead according to the manufacturer’s instructions as we described elsewhere^14^. After confirming the signal quality index to be greater than 95%, BIS and PSI were recorded from anesthesia induction to the end of anesthesia. BIS-EMG was the EMG value in the BIS monitor which was a logarithmic scale of total power in the 70–110 Hz range, averaged over the preceding 10 s. PSI-EMG was the EMG value in the PSI monitor which was a weighted percentage of half-epochs (over the past one minute) in which muscle activity, as measured by the power in the β2 band (25–50 Hz range), exceeds a threshold level. The TOF-Watch SX (Organon Ltd., Dublin, Ireland) was applied at the right or left adductor pollicis to monitor NMB. After 5 s of 50-Hz tetanic stimulus, the TOF-Watch SX was calibrated using automated CAL2 mode. In addition, 2 mg kg^−1^ of propofol and 0.7 mg kg^−1^ of rocuronium were used for induction. We performed tracheal intubation after confirming a train of four (TOF) count of 0. Mechanical ventilation was adjusted to maintain 35–40 mmHg end-tidal carbon dioxide with a mixture of oxygen and medical air (Fraction of inspired oxygen: 0.5). Anesthesia was maintained using sevoflurane and adjusted to maintain BIS between 40 and 60. Every 15 s, the ulnar nerve was stimulated supramaximally through surface electrodes with TOF mode. When the TOF count reached two or higher during surgery, we injected 0.15 mg kg^−1^ of rocuronium to maintain the count under two.

### Study protocols

At the end of the operation, 0.5 μg kg^−1^ of fentanyl was injected for postoperative pain control. We adjusted the concentration of sevoflurane to maintain BIS in the range of 40–50 for the 10 min of the ‘stabilization period.’ After reaching a steady state of sevoflurane anesthesia and confirming a TOF count of 1 or 2, 2 mg kg^−1^ of sugammadex (baseline, T_0_) was injected. The end-tidal concentration of sevoflurane was maintained until the end of the 10-min study period (T_10_). The numerical values of BIS, BIS-EMG, PSI, and PSI-EMG, any clinical signs of arousal (i.e., eye-opening, spontaneous movement, cough, response to simple orders, complete recovery), and Modified Observers Assessment of Alertness and Sedation Scale were recorded at one-minute intervals for 10 min after sugammadex administration. The time from the T_0_ to the time of TOF ratio ≥ 90% (T_90%_) was also recorded. In case of incomplete reversal of NMB at T_10_ (TOF ratio < 90%), we administered an additional 2 mg kg^−1^ of sugammadex as a rescue medication. When confirming the completion of the study and reversal of NMB (TOF ratio ≥ 90%), sevoflurane was discontinued. The patients’ tracheas were extubated and transferred to the post-anesthetic care unit.

### Outcomes

The primary outcome was the difference between the changes in BIS and PSI from T_0_ to T_90%_ after sugammadex administration in steady-state sevoflurane anesthesia. Secondary outcomes were as follows: the differences between the changes in BIS and PSI from T_0_ to their maximum values (BIS_max_ and PSI_max_, respectively) during the 10-min study period; the differences between the changes in BIS-EMG and PSI-EMG from T_0_ to the T_90%_ and from T_0_ to their maximum values during the 10-min study period; the pattern of change and correlation between BIS and PSI values over time, which were recorded at one-minute intervals for 10 min after sugammadex administration; the correlation between the BIS and BIS-EMG values, PSI and PSI-EMG values, TOF ratio and BIS-EMG values, and TOF ratio and PSI-EMG values over time, which were recorded at one-minute intervals for 10 min after sugammadex administration.

### Statistical analysis

The sample size was calculated based on our pilot study (unpublished), which included 14 patients and the magnitudes of changes in BIS and PSI from T_0_ to T_90%_ were compared. The mean (standard deviation [SD]) changes in BIS and PSI were 5.8 (8.9) and 2.1 (1.4), respectively, after the administration of sugammadex. An interim power analysis t-test (a = 0.05) showed that 45 patients would be required to reveal a statistically significant difference with 80% power. Considering the 10% drop-out rate, we decided to recruit at least 50 patients.

The categorical variables are presented as numbers and percentages (%). Continuous variables were presented as mean (SD) or median (interquartile range [IQR]) as appropriate. The normality of continuous variables was assessed with the Shapiro–Wilk test. We compared the change between the BIS and PSI from T_0_ to T_90%_ after sugammadex administration using a t-test or Mann–Whitney U test. In addition, a paired t-test or Wilcoxon signed-rank test was used to compare the baseline values of BIS, BIS-EMG, PSI, and PSI-EMG with their values at T_90%_ or their maximum values during the 10-min study period. The pattern of change between BIS and PSI values over time, which were recorded at one-minute intervals for 10 min after sugammadex administration, were compared using the Generalized Estimating Equation (GEE). Pearson’s correlation analysis was used to analyze the relationships between BIS and PSI, BIS and BIS-EMG, PSI and PSI-EMG, TOF ratio and BIS-EMG, and TOF ratio and PSI-EMG over time.

Statistical analyses were performed using SAS version 9.4 (SAS Institute, Cary, NC, USA) and SPSS version 27.0 (IBM Corp., Armonk, NY, USA). P-values less than 0.05 were considered statistically significant.

## Results

**Figure 1:**
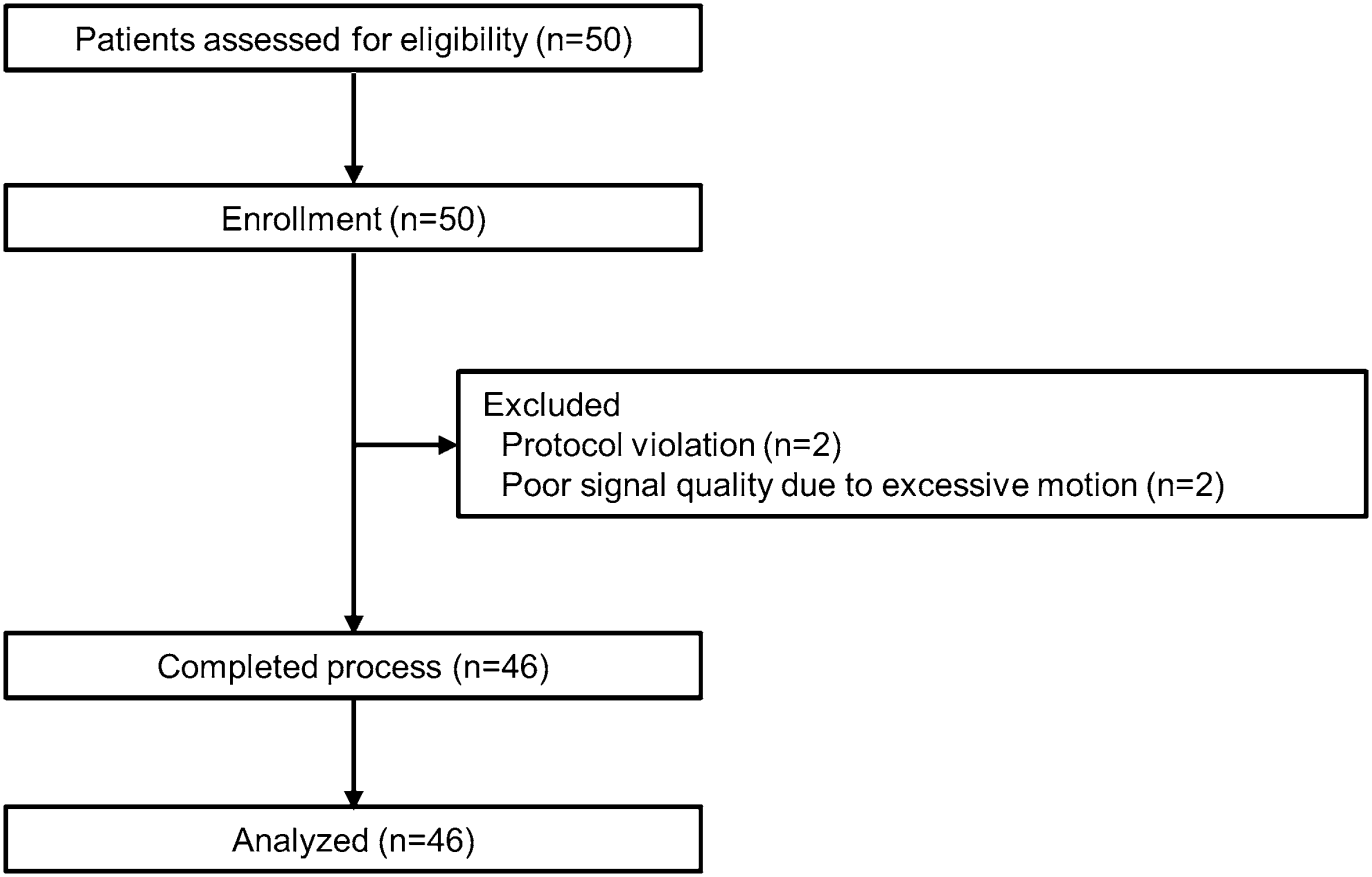
CONSORT diagram.

A total of 50 patients was recruited for this study. Two cases of protocol violation with a TOF count greater than 2 before sugammadex administration and additional two cases that exhibited excessive movement were excluded from the analysis because of poor signal quality and missing values in BIS and PSI, but they were included in the incidence calculation of clinical signs of arousal. A final total of 46 patients completed the study protocols and was analyzed (Fig. 1).

Baseline characteristics and intraoperative data are presented in Table 1. The changes in BIS and PSI values from T_0_ to T_90%_ were not significantly different (median difference 0; 95% confidence interval [CI] − 1.5 to 2; P = 0.81), neither were the changes in BIS and PSI values from T_0_ to their maximum values (median difference, 1.5; 95% CI − 0.5 to 3; P = 0.16). The medians (IQR) time from T_0_ to T_90%_ and maximum BIS were 5 (3–7) minutes and 6 (2–9) minutes, respectively. The BIS and PSI values at T_0_ and T_90%_, and their maximum values are presented in Table 2. The changes in BIS and PSI from T_0_ to T_90%_ were not significant, but the changes from baseline to BIS_max_ and PSI_max_ values were statistically significant (median difference 6; 95% CI 4–9; P < 0.001 and median difference 5; 95% CI 3–6; P < 0.001, respectively). The magnitudes of changes from T_0_ to T_90%_ were not significantly different between the BIS-EMG and PSI-EMG values (median difference 0; 95% CI 0–0; P = 0.41), neither were the changes from T_0_ to their maximum values (median difference 0; 95% CI 0–0; P = 0.87).

**Table 1:** Baseline characteristics and intraoperative data.

Variable	n = 46
Male	25 (54.4)
Age	46.5 [28.3–54.0]
Body mass index (kg/m^2^)	24.5 (2.5)
ASA PS I/II	22/24 (47.8/52.2)
Surgical procedure	
Abdominal	14 (30.4)
Orthopedic	29 (63.0)
Urological	3 (6.5)
Duration of surgery (min)	93.0 [46.3–121.0]
Duration of anesthesia (min)	129.0 [88.3–176.8]
Total amount of rocuronium (mg)	53.7 [44.0–63.8]
Total amount of remifentanil (mg)	0.0 [0.0–0.0]
Time to TOFR 0.9 (min)	5.0 [3.0–7.0]
Time to maximal BIS (min)	5.5 [2.0–9.0]
EtSevo (Vol%) at steady state	2.0 [1.6–2.3]

Data expressed as mean (SD), median [IQR], or n (%).

*ASA PS* American Society of Anesthesiologists' physical status, *TOFR* train of four ratio, *BIS* bispectral index, *EtSevo* end-tidal sevoflurane concentration.

**Table 2:** Comparison of data before and after administration of sugammadex.

	Baseline (T0)	TOFR 0.9		Maximal BIS and PSI values	
			*P* value*		*P* value**
BIS	44 [41–46]	44 [40–47]	0.75	50 [45–54]	< 0.001
BIS-EMG	32 [29–42]	34 [29–43]	0.03	35 [29–44]	0.02
PSI	32 [28–36]	32 [27–37]	0.92	36 [34–40]	< 0.001
PSI-EMG	0 [0–0]	0 [0–0]	0.07	0 [0–0]	0.01

Data expressed as median [IQR].

*The *P* value for the baseline vs. BIS and PSI values at the time of TOFR ≥ 0.9

**The *P* value for the baseline vs. Maximal BIS and PSI values.

*TOFR 0.9* Train of four ratio of 0.9, *BIS* bispectral index, *PSI* patient state index, *BIS-EMG* electromyography recorded by the BIS electrode, *PSI-EMG* EMG recorded by the PSI electrode.

**Figure 2:**
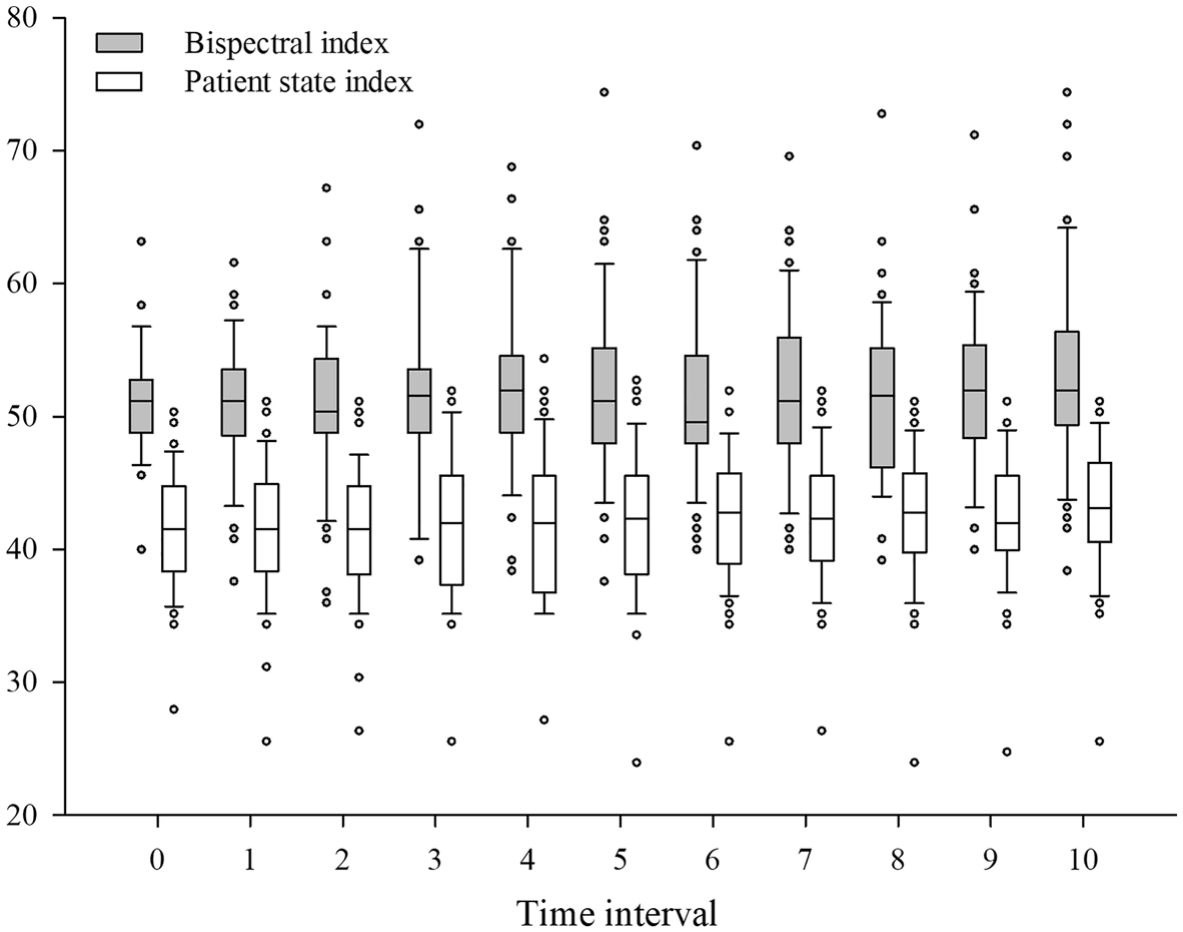
Box and whisker plots of bispectral index and patient state index during the 10-min study period. Sugammadex was given at time point 0. The boxes represent the median values and the 25th and 75th percentiles. The whiskers represent the minimum and maximum values, excluding outliers. The points represent the outliers.

**Figure 3:**
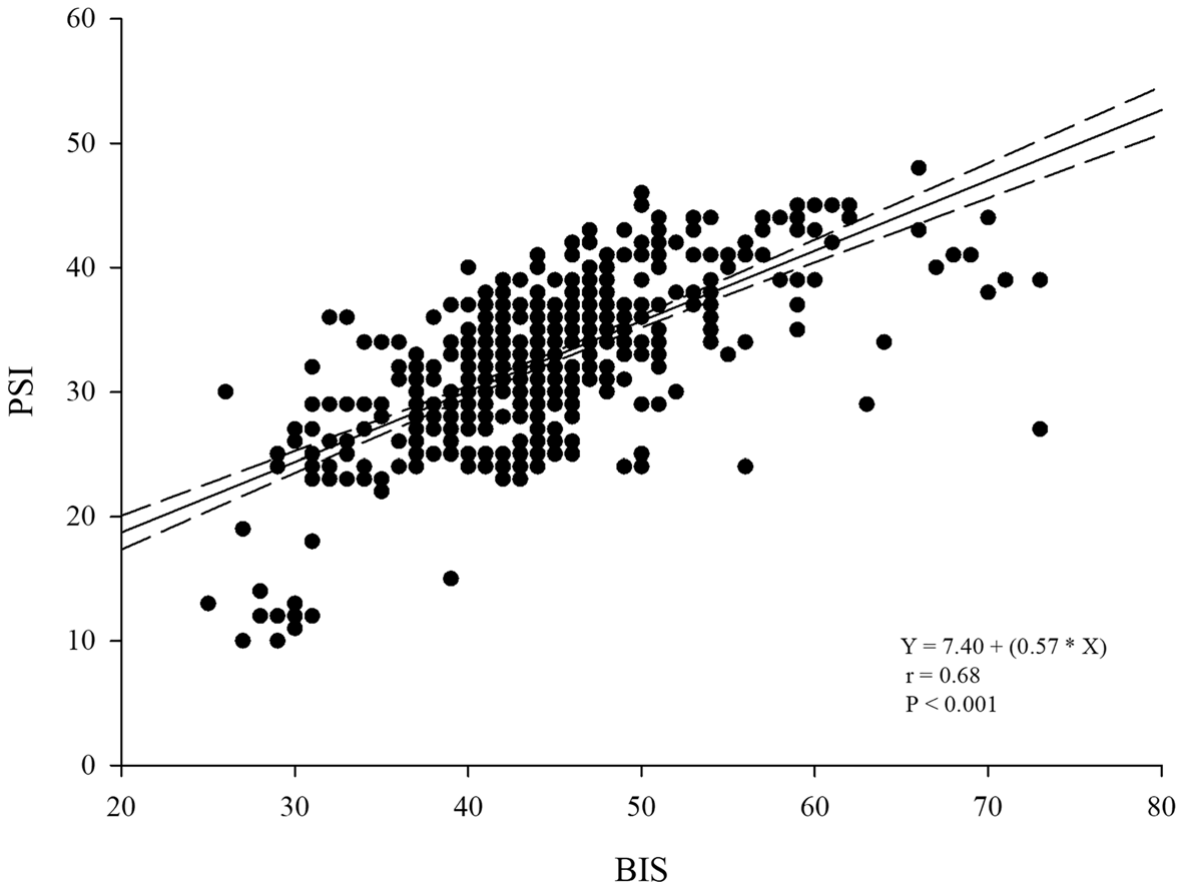
Correlation between bispectral index (BIS) and patient state index (PSI) values simultaneously recorded during 10 min after sugammadex administration.

**Figure 4:**
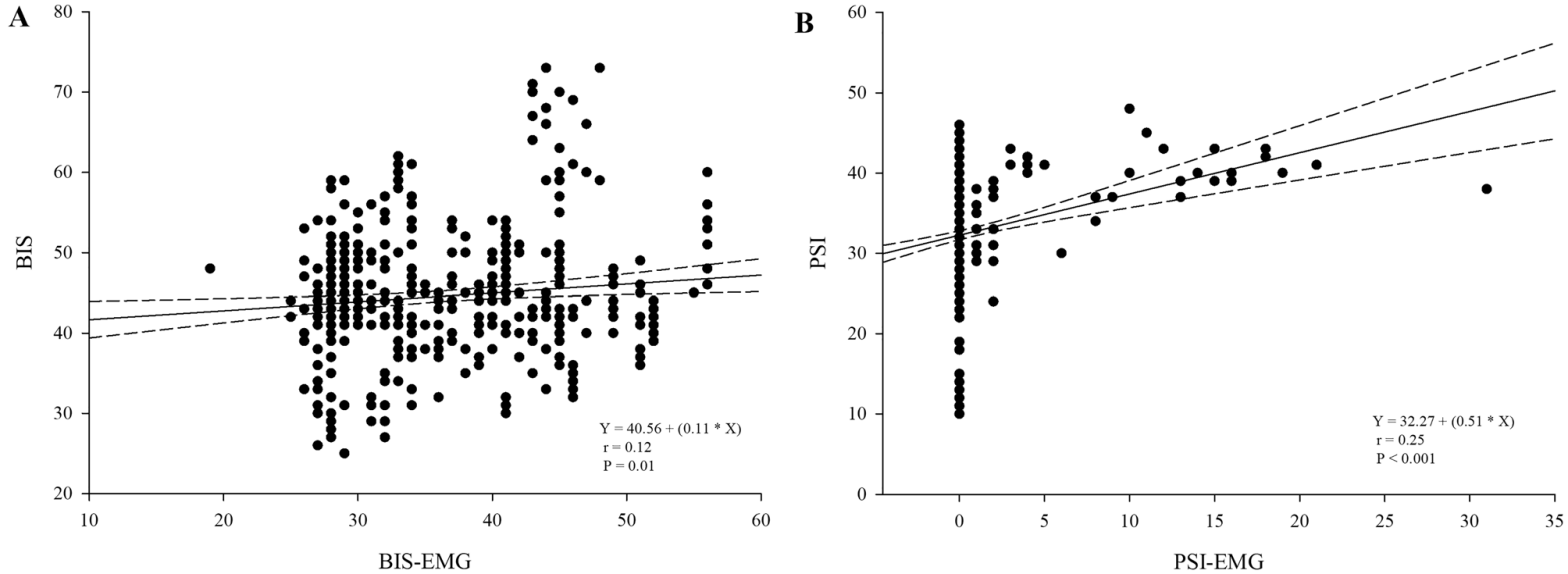
Correlations between each parameter and their electromyography (EMG) values during 10 min after sugammadex administration. (**A**) correlation between bispectral index (BIS) and BIS-EMG values, (**B**) correlation between patient state index (PSI) and PSI-EMG values. *BIS-EMG* EMG recorded by the BIS electrode, *PSI-EMG* EMG recorded by the PSI electrode.

Figure 2 showed the distributions of BIS and PSI every minute for 10 min of the study period. GEE showed that the patterns of change over time were not significantly different between the BIS and PSI (P = 0.24). The BIS and PSI values during steady-state sevoflurane anesthesia after sugammadex administration exhibited a positive correlation (correlation coefficient [r] = 0.684, P < 0.001) (Fig. 3). BIS and BIS-EMG (r = 0.12, P = 0.01), as well as PSI and PSI-EMG (r = 0.25, P < 0.001) demonstrated weak positive correlations (Fig. 4).

One patient who did not completely recover from neuromuscular blockade (TOF ratio < 0.9) after the 10-min study period received an additional 2 mg kg^−1^ of sugammadex and completely recovered after 15 min. We observed that four patients exhibited unexpected movements and coughing after injection of sugammadex, along with BIS elevation. MOAA/S was 0 in all patients and no patient experienced awareness, recall, or adverse effects during the study period.

## Discussion

This comparative study demonstrated similar magnitudes and patterns of changes in BIS and PSI induced by sugammadex reversal of NMB under steady-state sevoflurane anesthesia. Sugammadex administration increased BIS and PSI, which exhibited a positive correlation with each other. BIS-EMG and PSI-EMG values also increased significantly. Moreover, weak positive correlations existed between BIS and BIS-EMG and between PSI and PSI-EMG.

In the current study, sugammadex administration was associated with increased BIS and PSI values. Several studies demonstrated that the reversal of NMB with sugammadex also increased BIS^4–7^. There are mainly two explanations for this increase. Fast electrical rhythms in the γ range (30–100 Hz) recorded from the scalp are inducible not only by mental activity but also by EMG signal that is unrelated to the cognitive effect^16^. Owing to the overlapping frequencies of EEG and EMG, EEG-based monitoring devices for depth of anesthesia could confuse changes in EMG signal with the patient’s state of consciousness during recovery from general anesthesia^9,10^. In this regard, our study results could be partially explained by EMG artifacts because BIS and PSI increased along with frontalis EMGs. However, the BIS and PSI only had weak correlations with their EMG values, 35 patients had PSI-EMG values of 0 reflecting no EMG activity, and four of the study subjects exhibited unexpected movements. The changes in BIS and PSI during sugammadex reversal of NMB might be attributable to actual arousal induced by increased afferent signals and the unexpected movements could be considered evidence of awakening as in the previous studies^4,17–19^. The afferentation theory of cerebral arousal posits that agents or maneuvers that produce muscle stimulation will activate the sensory receptors in muscle spindles and generate action potentials that are transmitted by peripheral nerves to the dorsal spinal cord, which eventually lead to cerebral arousal^8,11^. While the patients are paralyzed, neuromuscular blocking agents abolish movement and muscle afferent response and also attenuate the EEG responses. Once these patients are reversed with sugammadex, an abrupt increase in afferent signals from the muscle spindle is produced, which stimulates the arousal center in the central nervous system to induce arousal^8,11,12^. Clear evidence of wakefulness and awareness including eye-opening and response to simple commands has been reported after sugammadex administration while propofol was maintained at a constant dosage^4^. Unfortunately, our study design could not clarify the cause of the increases in BIS and PSI. Further research is warranted to determine which of the two mechanisms was the main culprit of increases in the BIS and PSI values after sugammadex administration.

Despite major differences in their algorithms, we observed similar changes in the BIS and PSI values after sugammadex administration under sevoflurane anesthesia, which was consistent with our previous study under propofol-remifentanil intravenous anesthesia^14^. In addition, BIS-EMG and PSI-EMG demonstrated similar increases during a sugammadex reversal of NMB and both values had weak positive correlations with BIS and PSI, respectively. Considering these results, we could not conclude which parameter was less disturbed by EMG interference. If the BIS or PSI values increased without activation of the EMG signal after the sugammadex reversal of NMB, we could consider it as actual arousal. In contrast, if the BIS or PSI values increased along with EMG activity, but without clinical signs of arousal, we should consider the possibility of false elevation induced by EMG artifacts, rather than actual arousal.

In the current study, the time from T_0_ to T_90%_ was longer and the increases in BIS and PSI were smaller than in our previous study with a similar setting under propofol-remifentanil anesthesia^14^. We attributed this difference to the type of anesthetics—inhalational or intravenous anesthetics. Volatile anesthetics augment the potency of nondepolarizing muscle relaxants by enhancing antagonist affinity at the receptor site NMB^20–22^. It caused slower recovery of NMB after sugammadex and decreased changes in PSI and BIS at T_10_ as in the case of gradual reversal of NMB by neostigmine^5^. In addition, the incidence of arousal signs in this study (4/50, 8%) was lower than 19–27% of other studies^4,17,19^. Although the lower incidence could be attributable to fentanyl^23^, inhalational anesthetics might reduce the incidence because it could prevent an abrupt increase in muscle afferent activity by sugammadex and reduce the cerebral arousal response^8,11^.

The recommended ranges for general anesthesia are 40–60 for BIS and 25–50 for PSI^24^. Among 46 patients who did not show any clinical signs of arousal or recall in the current study, six cases exhibited BIS values out of range for general anesthesia (> 60) and two of them exhibited BIS values above 70 which could be regarded as a clinical state where a patient can respond to loud verbal or limited tactile stimulation^25^. In contrast, none of these patients showed out-of-range values in PSI. If we monitored BIS only, they could be confused to be awake leading to unnecessary deepening of anesthesia for fear of awareness or hasty extubation, which could cause potential respiratory complications including desaturation, laryngospasm, stridor, bronchospasm, and reintubation^26^. In this regard, PSI might be a more stable parameter appropriately reflecting the actual depth of hypnosis in the situation with an abrupt increase of EMG activity.

There are several limitations in the current study. First, we could not discriminate the cause of increases in BIS and PSI. We only suggest that the observed increases in BIS and PSI might not be strictly associated with actual arousal as their values demonstrated positive correlations with their EMG values. In addition, we did not consider the effect of fentanyl on EEG. However, the doses of fentanyl (0.5 μg/kg bolus) were too small to affect EEG^27–29^. Third, our study design did not allow us to compare the influences according to the type of reversal agent. Neostigmine or pyridostigmine has a gradual recovery profile and could exhibit differences in BIS and PSI during the reversal of NMB.

In conclusion, sugammadex administration increased BIS and PSI under steady-state sevoflurane anesthesia, which demonstrated similar magnitudes and patterns of changes. These indices correlated weakly with their EMG values. Both PSI and BIS values can be affected by EMG artifacts after sugammadex administration.

## Data Availability

The dataset generated during the current study is available from the corresponding author upon request.
